# A Diagnostic Puzzle: Unveiling Tuberculosis Peritonitis in an Immunocompromised Patient

**DOI:** 10.7759/cureus.93962

**Published:** 2025-10-06

**Authors:** André Pereira, Inês Peixoto, Ana Silva, Rosa Cardoso, Helena Sarmento

**Affiliations:** 1 Internal Medicine, Unidade Local Saúde Alto Ave, Guimarães, PRT; 2 General Surgery, Unidade Local Saúde Alto Ave, Guimarães, PRT; 3 Pathology, Laboratório Unilabs, Porto, PRT

**Keywords:** abdominal tuberculosis, adenosine deaminase, anti-tuberculous therapy, extrapulmonary tuberculosis, hiv, tuberculous peritonitis

## Abstract

Tuberculosis (TB) remains a leading cause of infectious morbidity and mortality worldwide. Extrapulmonary TB, particularly abdominal TB, is a diagnostic challenge due to its nonspecific presentation and often low sensitivity for conventional tests. Immunocompromised patients, such as those with human immunodeficiency virus (HIV), are at increased risk and may lack classic systemic symptoms, preventing timely diagnosis. A 61-year-old HIV-positive female on antiretroviral therapy and with an undetectable viral load presented with progressive abdominal distension, postprandial fullness, and altered bowel habits over two months. Physical examination revealed ascites and a right supraclavicular lymphadenopathy. Laboratory findings showed normocytic anemia, thrombocytosis, hypoalbuminemia, and elevated inflammatory markers. Computed tomography demonstrated large-volume ascites, bilateral pleural effusions, and signs of chronic liver disease. Ascitic fluid analysis revealed lymphocyte predominance with elevated adenosine deaminase (ADA) levels but was negative for *Mycobacterium tuberculosis* by cytology, culture, and polymerase chain reaction (PCR). A definitive diagnosis was established via laparoscopic peritoneal biopsy, which demonstrated granulomatous inflammation with PCR confirmation of *M. tuberculosis*. Quadruple anti-tuberculous therapy was started, and the patient was referred for specialized follow-up. The clinical course was favorable, resulting in complete resolution of the ascites. This case underscores the diagnostic difficulty of abdominal TB, especially in HIV-infected individuals who may lack typical symptoms. While ascitic fluid ADA measurement is a useful supportive test, peritoneal biopsy remains the gold standard in cases where fluid analysis is inconclusive. Early diagnosis and prompt initiation of anti-tuberculous treatment are essential to avoid complications and improve outcomes. Abdominal TB should be considered in the differential diagnosis of unexplained ascites, particularly in immunocompromised patients. Multimodal diagnostic evaluation, including invasive tissue sampling, is often required to establish the diagnosis and guide timely management.

## Introduction

Tuberculosis (TB) remains one of the leading infectious causes of morbidity and mortality worldwide, despite being a preventable and curable disease [1,2]. According to the World Health Organization (WHO), an estimated 10.8 million people developed TB in 2023, with 1.09 million deaths among HIV-negative individuals and an additional 161,000 deaths among those living with HIV [1].

The clinical presentation of TB is heterogeneous, influenced by the site of infection, the host's immune status, and the disease burden [2,3]. Extrapulmonary TB may manifest with more subtle or organ-specific symptoms, postponing prompt diagnosis [3].

Abdominal TB is a form of extrapulmonary TB that involves the gastrointestinal tract, peritoneum, intra-abdominal lymph nodes, and visceral organs, either singly or in combination [4-6]. This TB manifestation accounts for 1% to 3% of all TB cases worldwide and represents 6% to 13% of extrapulmonary TB cases, with rates varying by geography [5]. The condition typically arises from the reactivation of latent *Mycobacterium tuberculosis *infection or via hematogenous or lymphatic dissemination from a primary pulmonary focus [5,6].

In HIV-positive patients, abdominal TB is increasingly recognized, with reported gastrointestinal involvement ranging from 1.7% to 19.7% in China and up to 71% in symptomatic cohorts in sub-Saharan Africa. Yet, large-scale epidemiological data remain limited [7-9].

Timely diagnosis and initiation of standard anti-TB therapy are crucial to prevent complications such as intestinal obstruction, fistula formation, or perforation [5,10]. This case underscores the importance of considering abdominal TB in patients presenting with chronic or unexplained abdominal symptoms, especially among individuals with known risk factors.

## Case presentation

A 61-year-old autonomous Black female from a middle-to-low-income background, an immigrant from Angola, and living in Portugal for the past four years, presented to the emergency department with a two-month history of increased abdominal circumference, associated with postprandial fullness and decreased stool frequency. She denied fever, nausea, vomiting, anorexia, or weight loss.

Past medical history included a sexually acquired HIV-1 infection, on antiretroviral therapy (elvitegravir, emtricitabine, tenofovir alafenamide, and cobicistat) with irregular follow-up since 2019, and arterial hypertension. Her last immunovirological assessment in 2019 showed a CD4 count of 612/µL (21.16%) and an undetectable HIV-1 viral load. No history of alcohol or drug abuse.

On physical examination, the patient was hemodynamically stable and afebrile. A painless, right-sided, infracentimetric supraclavicular lymphadenopathy was noted. The abdomen was distended, with shifting dullness and a positive fluid wave test, evidence of ascites.

Laboratory evaluation revealed normocytic normochromic anemia, thrombocytosis, hypoalbuminemia, and elevated C-reactive protein (Table 1).

**Table 1:** Blood test results performed at hospital admission APTT, activated partial thromboplastin time; ALT, alanine aminotransferase; AST, aspartate aminotransferase; CRP, C-reactive protein; GGT, gamma-glutamyl transferase; INR, international normalized ratio; LDH, lactate dehydrogenase; MCHC, mean corpuscular hemoglobin concentration; MCV, mean corpuscular volume; WBC, white blood cells.

Test	Result	Reference Range
Hemoglobin (g/dL)	9.5	14.0–18.0
Hematocrit (%)	29.2	41–53
MCV (fL)	74.3	83–103
MCHC (g/dL)	32.5	32.0–36.0
WBC (×10^3^/µL)	5.0	4.8–10.8
Neutrophils (×10³/µL)	2.9	1.8–7.7
Eosinophils (×10³/µL)	0.0	0.00–0.49
Basophils (×10³/µL)	0.0	0.0–0.1
Lymphocytes (×10³/µL)	1.7	1.0–4.8
Monocytes (×10³/µL)	0.3	0.12–0.80
Platelets (×10³/µL)	467	150–350
Urea (mg/dL)	20	15–39
Creatinine (mg/dL)	0.65	0.55–1.02
Sodium (mEq/L)	139	135–136
Potassium (mEq/L)	4.7	3.5–5.1
Chloride (mEq/L)	101	95–105
Total Bilirubin (mg/dL)	0.58	0.3–1.2
Direct Bilirubin(mg/dL)	0.24	0.0–0.3
AST (U/L)	25	12–40
ALT (U/L)	10	7–40
GGT (U/L)	22	0–38
LDH (U/L)	241	120–246
CRP (mg/L)	138.1	<3.0
Albumin (g/dL)	2.8	3.4–5.0
Prothrombin time (sec)	14.3	8.4–14.4
INR	1.2	-
APTT (sec)	29.7	20.9–34.9

Abdominopelvic computed tomography (CT) demonstrated moderate bilateral pleural effusion, large-volume ascites, and imaging features suggestive of chronic liver disease (liver heterogeneity with left and caudate lobe hypertrophy).

Given the patient's presentation with new-onset ascites, the initial differential diagnoses included portal hypertension secondary to cirrhosis, peritoneal carcinomatosis, tuberculous peritonitis, and, less likely, pancreatic or cardiac causes. A diagnostic paracentesis was performed, yielding clear yellow peritoneal fluid, which was sent for cytological and microbiological analysis. Ascitic fluid analysis revealed 352 leukocytes/µL, with a predominance of mononuclear cells. Biochemical analysis showed elevated adenosine deaminase (ADA) and a low serum-ascites albumin gradient (SAAG: 0.9 g/dL) (Table 2). In view of the lymphocytic predominance, high ADA level, and low SAAG (findings suggestive of an exudative, non-portal hypertensive etiology), TB peritonitis became a strong diagnostic consideration given the HIV history. Accordingly, the ascitic fluid was subsequently sent for *Mycobacterium tuberculosis *polymerase chain reaction (PCR) testing.

**Table 2:** Ascitic fluid analysis ADA, adenosine deaminase; LDH, lactate dehydrogenase; PMN, polymorphonuclear cells; RBC, red blood cells; WBC, white blood cells.

Test	Result
Cell count	
RBC (µL)	1,600
WBC (µL)	352
PMN/mononuclear cells	15.6%/84.4%
Other nucleated cells	9
Biochemistry	
pH	7.52
Glucose (mg/dL)	69
Albumin (g/dL)	1.9
Total proteins (g/dL)	6.3
LDH (U/L)	220
ADA (U/L)	124.4

The patient was admitted for clinical monitoring and further etiological investigation.

On day one, the peripheral blood smear was unremarkable. Hematinic studies revealed folate deficiency and elevated ferritin levels (Table 3). Lymphocyte subset analysis revealed a CD4 count of 489/µL (40.32%), and the HIV-1 RNA level was undetectable (Table 4). By day four, cytological, microbiological, and PCR analyses for *M. tuberculosis *in the ascitic fluid were negative.

**Table 3:** Additional laboratory investigation TIBC, total iron-binding capacity; TSAT, transferrin saturation.

Test	Result	Reference range
Folate (ng/mL)	2.3	>5.38
Vitamin B12 (pg/mL)	612	211–911
Serum iron (µg/dL)	12	50–170
TIBC (µg/dL)	125	250–425
Transferrin (mg/dL)	77	250–380
TSAT (%)	9.6	15–50
Ferritin (ng/mL)	472.8	10–291

**Table 4:** Lymphocyte subset analysis

Lymphocyte Subset	Result	Reference Range
T cells (µL)	64.61% (786)	49.0–81.0% (527–2846)
CD4 T cells (µL)	40.32% (489)	28.0–51.0% (332–1642)
CD8 T cells (µL)	23.91% (290)	12.0–38.0% (170–811)
B cells (µL)	9.64% (118)	7–32% (78–899)
NK cells (µL)	25.38% (310)	6–29% (67–1134)
CD4/CD8 Ratio	1.69	-

On day five, upper gastrointestinal endoscopy revealed gastric antral mucosa with areas of erythema and a few scattered erosions, findings consistent with superficial chronic gastritis.

**Figure 1:**
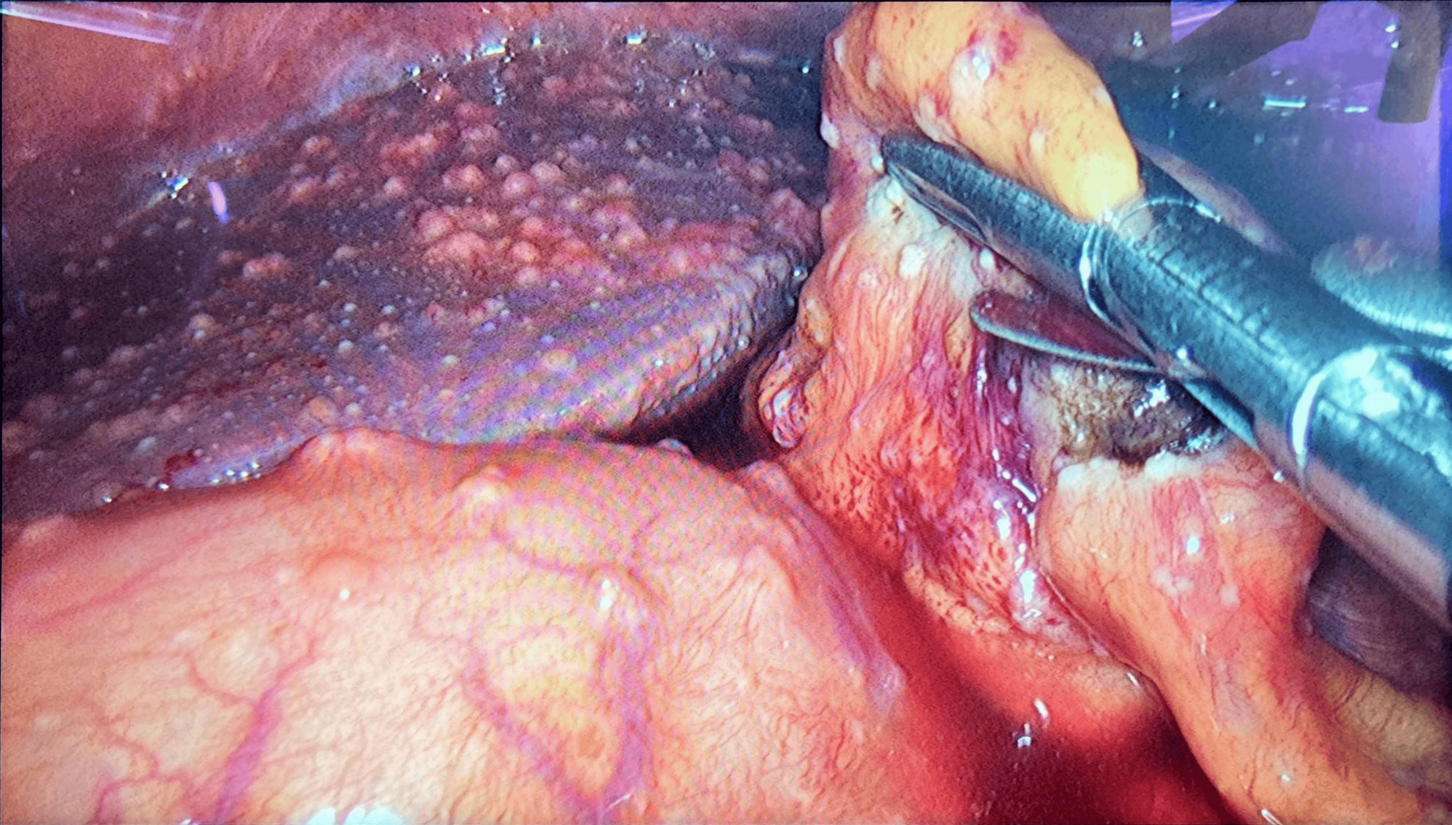
Laparoscopy Presence of multiple implants throughout the parietal peritoneum, greater *omentum*,* *and Glisson's capsule.

**Figure 2:**
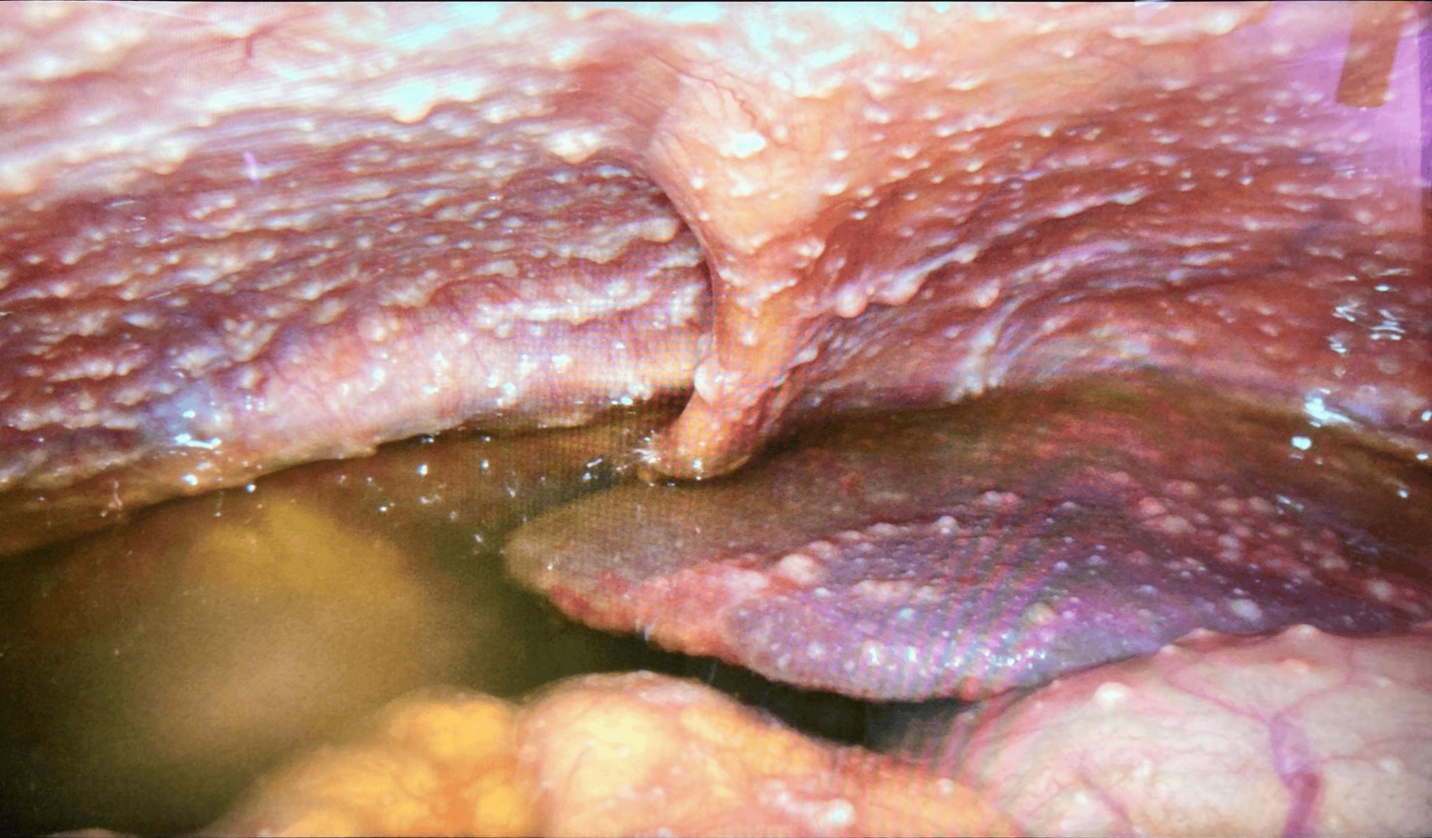
Laparoscopy Presence of multiple implants throughout the parietal peritoneum.

Given the inconclusive non-invasive workup, a laparoscopic peritoneal biopsy was performed on day nine, revealing multiple peritoneal implants disseminated throughout the abdominal cavity, including the *omentum *and intestinal loops (Figures 1, 2). Two tissue samples, one from the parietal peritoneum in the right hypochondrium and another from the greater *omentum *in the left hypochondrium, were submitted for histopathological examination and PCR testing for *M. tuberculosis*.

**Figure 3:**
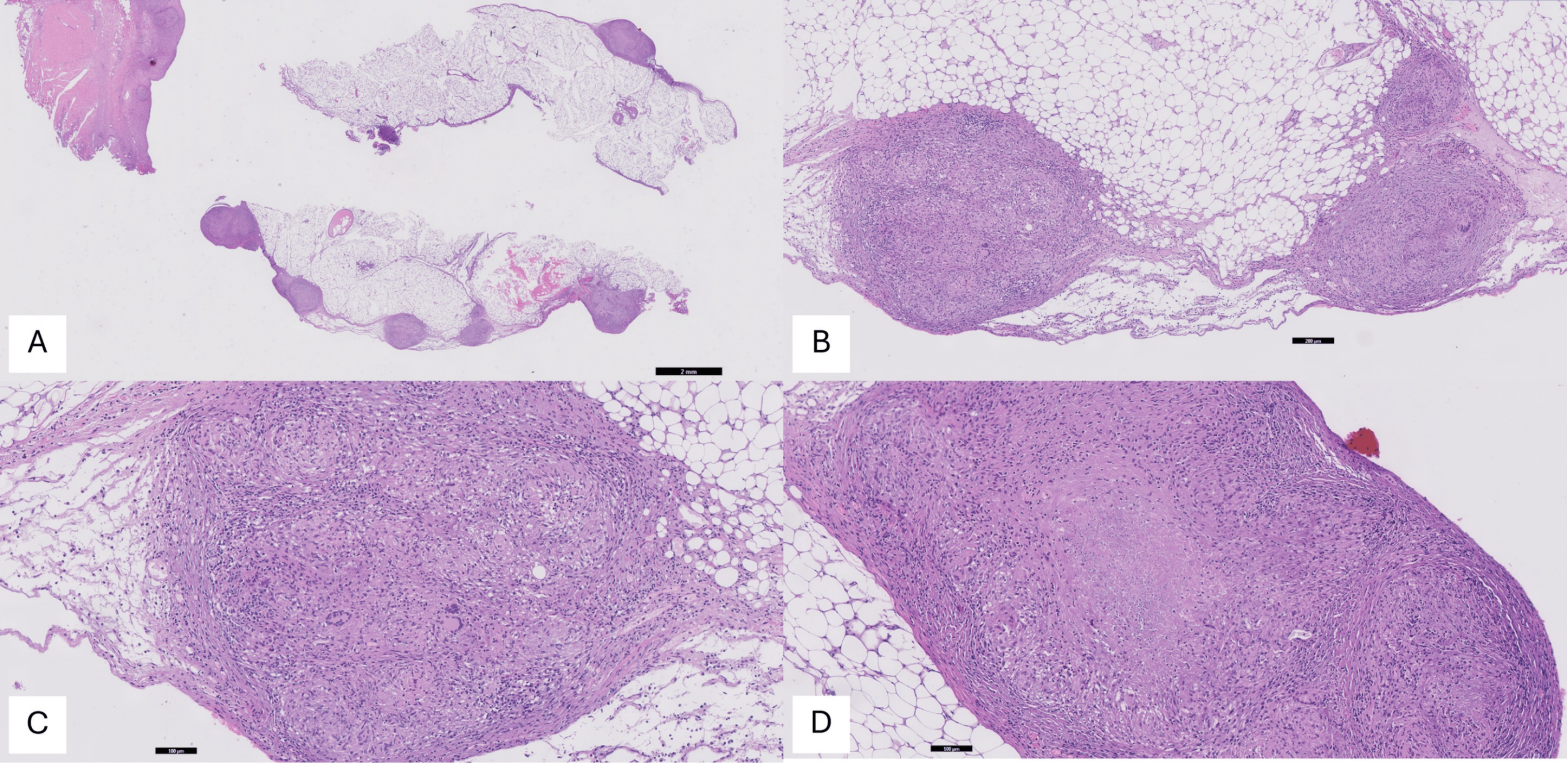
Light microscopy (hematoxylin/eosin) (A) Peritoneal adipose tissue exhibiting granulomatous inflammation (0.5x); (B) Peritoneal adipose tissue displaying three distinct fibrotic nodules, each containing centrally located granulomas with multinucleated Langhans-type giant cells, consistent with granulomatous inflammation (5x); (C) Histological section demonstrating fibrotic tissue containing granulomas with three multinucleated Langhans-type giant cells (10×); (D) Fibrotic tissue with peripheral aggregates of epithelioid histiocytes/macrophages exhibiting abundant cytoplasm. The central area shows necrosis, a hallmark feature of tuberculosis-associated granulomas (10×).

**Figure 4:**
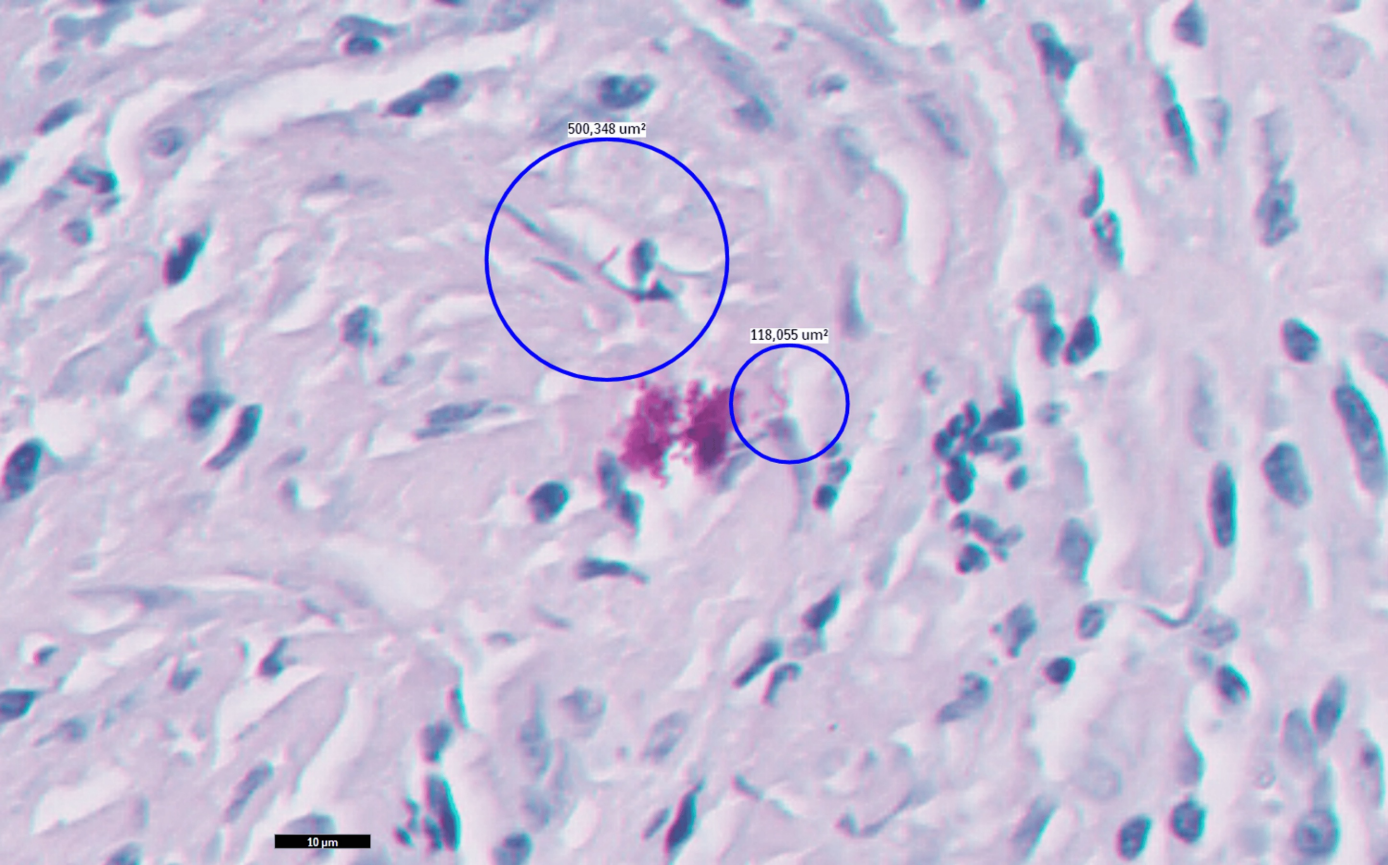
Light microscopy (Ziehl-Neelsen 100×) Red-stained bacilli observed on Ziehl-Neelsen staining, compatible with *Mycobacterium *species.

Quadruple anti-TB therapy was initiated, consisting of rifabutin, isoniazid, pyrazinamide, and ethambutol. PCR analysis of the peritoneal tissue confirmed the presence of *M. tuberculosis*. Histopathological findings from the peritoneal biopsy are illustrated in Figures 3, 4.

The patient was subsequently referred to a Pulmonology Diagnostic Center for continuation of anti-TB therapy and to an Infectious Diseases consultation for specialized follow-up. The clinical course was favorable, with complete resolution of the ascites after six months of therapy.

## Discussion

Abdominal TB remains a significant diagnostic challenge due to its highly variable clinical presentation and the frequent overlap of symptoms with other intra-abdominal conditions such as malignancies, chronic liver disease, and inflammatory bowel disorders [5-10]. Although pulmonary TB remains the most common manifestation, extrapulmonary involvement accounts for approximately 15-20% of TB cases globally [5]. Geographic variability plays an important role, as higher rates are observed in endemic regions, particularly among immunocompromised populations [1,5].

Building upon this epidemiological context, understanding the underlying mechanisms of abdominal TB is essential for accurate diagnosis. The pathogenesis of abdominal TB involves multiple potential routes of infection. The most widely accepted mechanisms include reactivation of latent *M. tuberculosis *infection, hematogenous dissemination from a primary pulmonary focus, and lymphatic spread from mesenteric nodes [4-6]. Less commonly, direct ingestion of infected sputum or contiguous extension from adjacent organs may contribute. Immunosuppressed individuals, particularly those living with HIV, have an increased susceptibility to extrapulmonary TB due to impaired cellular immunity [3,11]. In our patient, this factor likely played a key role, as HIV infection may have contributed both to disease development and to the atypical clinical presentation observed.

Given these pathophysiological mechanisms, diagnosis of abdominal TB is often delayed due to nonspecific clinical manifestations, such as abdominal distension, altered bowel habits, fever, and weight loss, which can mimic alternative diagnoses, including peritoneal carcinomatosis and cirrhosis [5,6]. In this case, the subacute onset of ascites and nonspecific gastrointestinal symptoms complicated the initial evaluation, leading to a broad differential diagnosis and necessitating a systematic diagnostic approach.

Ascitic fluid analysis remains a cornerstone in the evaluation of suspected TB peritonitis but is inherently limited by low sensitivity in paucibacillary disease [4-6,12]. Typical findings include a lymphocyte-predominant exudate with a low SAAG, as observed in our patient. Of the available biomarkers, ADA is considered one of the most reliable surrogate markers for TB peritonitis. A recent meta-analysis demonstrated that ADA levels above 100 U/L have high specificity for abdominal TB, making this a valuable diagnostic adjunct in clinical practice [12]. In our case, despite negative microbiological and PCR results, the markedly elevated ADA level (124.4 U/L) strongly supported the suspicion of TB peritonitis.

Abdominopelvic imaging is a critical adjunct in the evaluation of suspected abdominal TB. While not pathognomonic, it can demonstrate highly suggestive features, including peritoneal thickening, nodularity, ascites, omental caking, and intra-abdominal lymphadenopathy [5,10]. Such findings contribute to raising clinical suspicion and guiding further invasive testing, although imaging alone rarely establishes a definitive diagnosis [10].

When non-invasive tests are inconclusive, laparoscopic peritoneal biopsy becomes pivotal, with diagnostic yields exceeding 90% [4,5,10]. In our case, laparoscopic evaluation revealed multiple peritoneal implants, and histopathology demonstrated caseating granulomatous inflammation, while PCR confirmed the presence of *M. tuberculosis*, ultimately securing a conclusive diagnosis.

Importantly, HIV infection substantially modifies the clinical course and therapeutic approach in abdominal TB. In addition to increasing susceptibility, HIV often masks classic systemic features such as fever, night sweats, and weight loss, leading to delayed recognition [11]. Furthermore, therapeutic decisions are complicated by potential drug-drug interactions between rifamycins and antiretroviral therapy [2,5]. In this patient, careful regimen selection was required, and rifabutin was chosen instead of rifampicin to minimize these interactions, consistent with current recommendations [2,5].

Once the diagnosis is established, standard anti-TB therapy remains the mainstay of management. The standard six-month regimen, comprising a two-month intensive phase with isoniazid, rifamycin, pyrazinamide, and ethambutol followed by a four-month continuation phase with isoniazid and rifamycin, achieves high treatment success rates [2,5]. In cases of HIV co-infection, early initiation of antiretroviral therapy alongside TB treatment is associated with improved survival [11]. Our patient demonstrated complete clinical recovery, with resolution of ascites, following the prompt initiation of therapy, highlighting the importance of early recognition, individualized management, and timely treatment initiation.

## Conclusions

This case exemplifies the diagnostic complexity of abdominal TB, particularly in the context of HIV co-infection. While ascitic fluid ADA measurement is a valuable diagnostic adjunct, definitive diagnosis often requires peritoneal biopsy. Clinicians should maintain a high index of suspicion for abdominal TB in patients presenting with unexplained ascites and relevant risk factors. Early diagnosis and appropriate anti-TB therapy remain paramount to improving patient outcomes and preventing serious complications.
